# Echocardiographic Progression of Calcific Aortic Valve Disease in Patients with Preexisting Aortic Valve Sclerosis

**DOI:** 10.31083/j.rcm2410293

**Published:** 2023-10-17

**Authors:** Jasmin Shamekhi, Carina Uehre, Baravan Al-Kassou, Marcel Weber, Atsushi Sugiura, Nihal Wilde, Victor Mauri, Verena Veulemans, Malte Kelm, Stephan Baldus, Georg Nickenig, Sebastian Zimmer

**Affiliations:** ^1^Heart Center, Department of Medicine II, University Hospital Bonn, 53127 Bonn, Germany; ^2^Heart Center, Department of Cardiology, University Hospital Cologne, 50937 Cologne, Germany; ^3^Heart Center, Department of Cardiology, University Hospital Düsseldorf, 40225 Düsseldorf, Germany

**Keywords:** calcific aortic valve disease, CAVD, aortic valve stenosis, aortic calcification

## Abstract

**Background:**

We aimed to evaluate echocardiographic parameters to predict calcific aortic valve disease (CAVD) progression. CAVD ranges from aortic valve sclerosis (ASc) with no functional impairment of the aortic valve to severe aortic stenosis (AS). It remains uncertain, which patients with ASc have a particularly high risk of developing AS.

**Methods:**

We included a total of 153 patients with visual signs of ASc and peak flow velocity (Vmax) below 2.5 m/s at baseline echocardiography. Progression of CAVD to AS was defined as an increase in Vmax ≥2.5 m/s with a delta of ≥0.1 m/s; stable ASc was defined as Vmax below 2.5 m/s and a delta <0.1 m/s. Finally, we compared clinical and echocardiographic parameters between these two groups.

**Results:**

The mean age at baseline was 73.5 (± 8.2) years and 66.7% were of male sex. After a mean follow-up of 1463 days, 57 patients developed AS, while 96 patients remained in the ASc group. The AS group showed significantly more calcification (*p* < 0.001) and thickening (*p* < 0.001) of the aortic valve cusps at baseline, although hemodynamics showed no evidence of AS in both groups (ASc group: Vmax 1.6 ± 0.3 m/s versus AS group: Vmax 1.9 ± 0.3 m/s; *p* < 0.001). Advanced calcification (odds ratio [OR]: 4.8, 95% confidence interval [CI]: 1.5–15.9; *p* = 0.009) and a cusp thickness >0.26 cm (OR: 16.6, 95% CI: 5.4–50.7; *p* < 0.001) were independent predictors for the development of AS.

**Conclusions:**

The acquisition of simple echocardiographic parameter may help to identify patients with particularly high risk of developing AS.

## 1. Introduction

Calcific aortic valve disease (CAVD) is the most common valvular heart disease requiring interventional or surgical therapy in developed countries [1, 2]. CAVD ranges from aortic valve sclerosis (ASc) with no functional impairment of the aortic valve (AV) to severe aortic stenosis (AS) with hemodynamic impairment. Especially elderly patients are frequently affected and the prevalence of CAVD is increasing, due to global aging and more accurate diagnostic screening methods [3]. The initial stage of CAVD is characterized by visual signs of ASc without obstruction of the left ventricular outflow and is present in almost 30% of adults over 65 years of age [4]. Severe AS represents the end-stage of CAVD with hemodynamic compromise resulting in shortness of breath, loss of consciousness and/or chest pain due to obstruction of blood flow through the stenotic aortic valve. The prevalence of severe AS is about 3% in adults over 75 years of age [4, 5]. To date, there is no medical therapy available to prevent the progression of CAVD and it remains uncertain, which patients with ASc are at a particularly high risk of developing AS.

In this study, we evaluated the prevalence of CAVD progression in patients with pre-existent ASc and assessed echocardiographic parameters to predict disease progression and identify patients at a high risk of developing AS.

## 2. Materials and Methods

### 2.1 Study Design and Patient Population

**Fig. 1.**
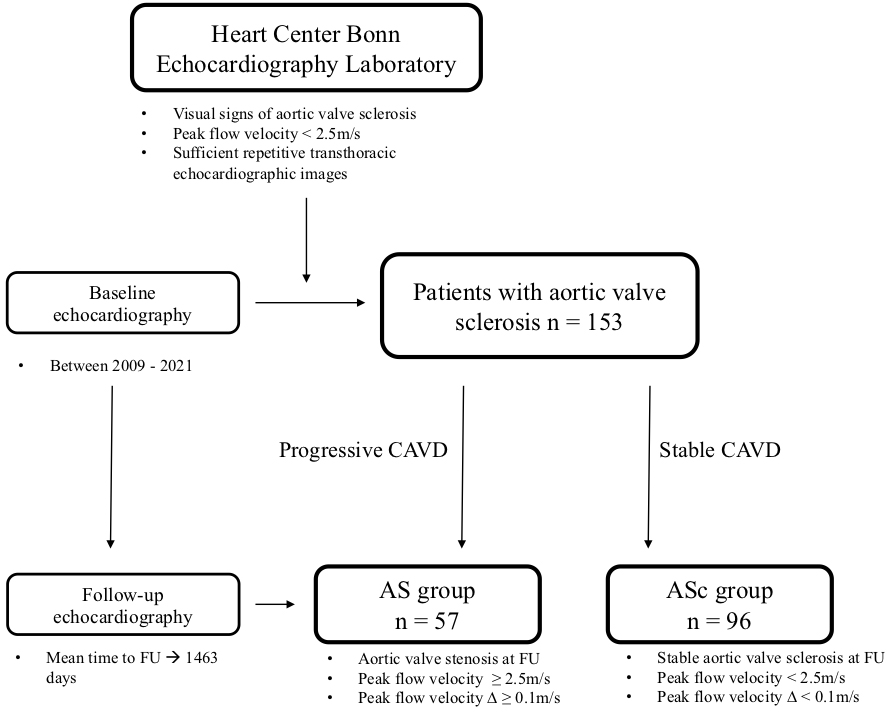
**Study flow chart. **AS, aortic stenosis; ASc, aortic valve sclerosis; CAVD, calcific aortic valve disease; FU, follow-up.

In this study, we compared clinical and echocardiographic parameters of patients with aortic valve sclerosis at baseline, who either developed aortic valve stenosis (mild, moderate or severe) during the follow-up echocardiography (AS group), or remained in the preceding stage with stable calcific aortic valve disease (ASc groups). The study design is shown in Fig. 1.

In detail, the database of the echocardiography laboratory of the Heart Center Bonn, which is a consecutive patient data registry, was retrospectively analyzed for patients with signs of aortic valve sclerosis without functional impairment of the aortic valve, defined as peak flow velocity below 2.5 m/s in transthoracic echocardiography. Prerequisite for the inclusion to the study was the availability of repetitive echocardiographic images (at least two) to evaluate the progression of CAVD over time. Exclusion criteria were missing or incomplete echocardiographic images at baseline or follow-up. Patients with aortic valve prostheses or bicuspid aortic valves were also excluded from the analysis. The presence of aortic valve sclerosis was assessed by an experienced physician. Progression of CAVD was defined as an increase of peak flow velocity ≥2.5 m/s with a delta of at least 0.1 m/s (Δ ≥0.1 m/s); stable CAVD complied with a peak flow velocity below 2.5 m/s and a delta <0.1 m/s.

The primary endpoint was the progression of calcific aortic valve disease to any stage of AS. Clinical and echocardiographic parameters including CAVD stage were assessed at follow-up and used to assign patients into two groups according to disease progression: patients with stable calcific aortic valve disease (ASc group) and patients with any stage of aortic valve stenosis (AS group) in the follow-up echocardiography. For the statistical analysis, we compared baseline and echocardiographic parameters between these two groups and evaluated their predictive value for the development of aortic valve stenosis.

### 2.2 Echocardiographic Parameters

Transthoracic echocardiography is still the method of choice for the diagnosis and evaluation of aortic valve stenosis [6]. The following echocardiographic parameters were assessed and evaluated in this study: left ventricular outflow tract (LVOT) diameter, diameter of the aortic root and the ascending aorta, thickness of the left- (LCC), right- (RCC), and non-coronary cusp (NCC) (measured at the thickest point of the respective cusp), the aortic valve area (AVA) as calculated by continuity equation and measured by planimetry, the mean pressure gradient (MPG) of aortic valve, the maximum pressure gradient (maxPG) of aortic valve, the aortic valve peak flow velocity (AV Vmax), the time to peak velocity, the stroke volume, the systolic duration, the degree of aortic valve regurgitation, visual signs of calcification (divided into minor and major calcification as a binary parameter) and reduced mobility of the left-, right-, and non-coronary cusp (binary variable with the categories “yes” and “no”, respectively), the degree of mitral valve regurgitation, left ventricular hypertrophy, the diastolic and systolic interventricular septal thickness, the degree of diastolic dysfunction, the E/e’ ratio, the left ventricular ejection fraction (LVEF), the left ventricular end-diastolic and end-systolic volume, and the left atrial end-diastolic and end-systolic volume. All echocardiographic parameters were assessed in accordance with the recommendations from the American Society of Echocardiography [7].

### 2.3 Statistical Analysis

Data are presented as mean ± standard deviation, if normally distributed, or as median and an interquartile range (IQR) (quartile 1/quartile 3), if not normally distributed. Continuous variables were tested for having a normal distribution by using the Kolmogorov-Smirnov test. Categorical variables are given as frequencies and percentages. For continuous variables, a Student’s *t* test or a Mann-Whitney *U* test-was performed for comparison between two groups. When comparing more than two groups, analysis of variance (ANOVA) or the Kruskal-Wallis test was used. Spearman’s correlation coefficients were used to assess associations. The χ^2^ test was used for analysis of categorical variables. To evaluate the prognostic value of aortic valve cusp thickness for the prediction of disease progression, receiver-operating characteristic (ROC) curves were generated to determine the optimum cut-off value. In consideration of the Youden-Index (Youden-Index = 0.64), a cusp thickness >0.26 cm was used for statistical analysis. Finally, we performed a multivariate regression analysis, which included univariate predictors with a *p*-value < 0.05, and a ROC curve analysis to assess independent predictors for the progression of CAVD.

Statistical significance was assumed when the null hypothesis could be rejected at *p* < 0.05. Statistical analyses were conducted with IBM SPSS Statistics version 27.0.0.0 (IBM Corporation, Somers, NY, USA). The investigators initiated the study, had full access to the data, and wrote the manuscript. All authors vouch for the data and its analysis.

## 3. Results

### 3.1 Overall Study Population

We identified 153 patients eligible to be included in the study. Clinical and echocardiographic parameters are shown in Table 1.

**Table 1.** **Clinical and echocardiographic parameters of the overall study population**.

All patients		
(n = 153)		
Clinical parameters		
Age, ± SD (years)		73.5 ± 8.2
BMI, ± SD (${\mathrm{kg/m}}^{2}$)		27.0 ± 4.6
Male sex, n (%)		102 (66.7)
PAD, n (%)		19 (12.4)
CKD, n (%)		37 (24.2)
Dialysis, n (%)		8 (5.2)
Hypertension, n (%)		132 (86.3)
Diabetes, n (%)		34 (22.2)
Dyslipidemia, n (%)		87 (56.9)
Smoker, n (%)		47 (30.9)
Atrial fibrillation, n (%)		76 (49.7)
History of CAD, n (%)		90 (58.8)
Previous stroke, n (%)		15 (9.8)
MAPT, n (%)		51(33.6)
DAPT, n (%)		25 (16.3)
OAC/DOAC, n (%)		79 (51.6)
Echocardiographic parameters at baseline		
AVA by continuity equation, ${\mathrm{cm}}^{2}$		1.9 ± 0.7
MPG, mmHg		6.7 ± 3.0
maxPG, mmHg		13.0 ± 5.6
Vmax, m/s		1.7 ± 0.4
Aortic regurgitation, n (%)		
	Grade 0	71 (46.4)
	Grade I	65 (42.5)
	Grade II	17 (11.1)
	Grade III	-
Mitral regurgitation, n (%)		
	Grade 0	15 (9.8)
	Grade I	91 (59.5)
	Grade II	42 (27.5)
	Grade III	5 (3.3)
Ejection fraction, %		53.7 ± 11.8
Aortic stenosis, n (%)		
	None	153 (100)
	Mild	-
	Moderate	-
	Severe	-
Echocardiographic parameters at follow-up		
Time to follow-up, days		1463 ± 953
Aortic stenosis at follow-up, n (%)		
	None	96 (62.7)
	Mild	19 (12.4)
	Moderate	29 (19.0)
	Severe	9 (5.9)
AVA by continuity equation, ${\mathrm{cm}}^{2}$		1.6 ± 0.8
MPG, mmHg		12.1 ± 9.8
maxPG, mmHg		23.6 ± 17.7
Vmax, m/s		2.3 ± 0.8
Aortic regurgitation, n (%)		
	Grade 0	67 (43.8)
	Grade I	73 (47.7)
	Grade II	13 (8.5)
	Grade III	-
Mitral regurgitation, n (%)		
	Grade 0	8 (5.2)
	Grade I	93 (60.8)
	Grade II	49 (32.0)
	Grade III	3 (2.0)
Ejection fraction, %		54.5 ± 11.7

Values are displayed as mean (± SD), median (IQR 1/3) or n (%). AVA, aortic valve area; BMI, body mass index; CAD, coronary artery disease; CKD, chronic kidney disease; DAPT, dual antiplatelet therapy; DOAC, direct oral anticoagulant; maxPG, maximum pressure gradient; MAPT, mono antiplatelet therapy; MPG, mean pressure gradient; OAC, oral anticoagulant; PAD, peripheral artery disease; Vmax, peak flow velocity; IQR, interquartile range.

The mean age of the overall study cohort was 73.5 ± 8.2 years and 66.7% of the patients were male. Most patients (86.3%) presented with arterial hypertension, 22.2% had diabetes, 56.9% suffered from dyslipidemia and 30.9% were active smokers. Almost two-thirds of the patients had concomitant coronary artery disease. Chronic kidney disease (CKD) was present in 24.2% of the patients, whereof 5.2% had terminal dialysis-dependent renal insufficiency. In the baseline transthoracic echocardiography, the mean AV Vmax was 1.7 ± 0.4 m/s, the MPG was 6.7 ± 3.0 mmHg and the mean AVA was 1.9 ± 0.7 ${\mathrm{cm}}^{2}$. The mean LVEF was 53.7 ± 11.8% and 53.6% of the patients suffered from mild to moderate concomitant aortic regurgitation (AR).

**Fig. 2.**
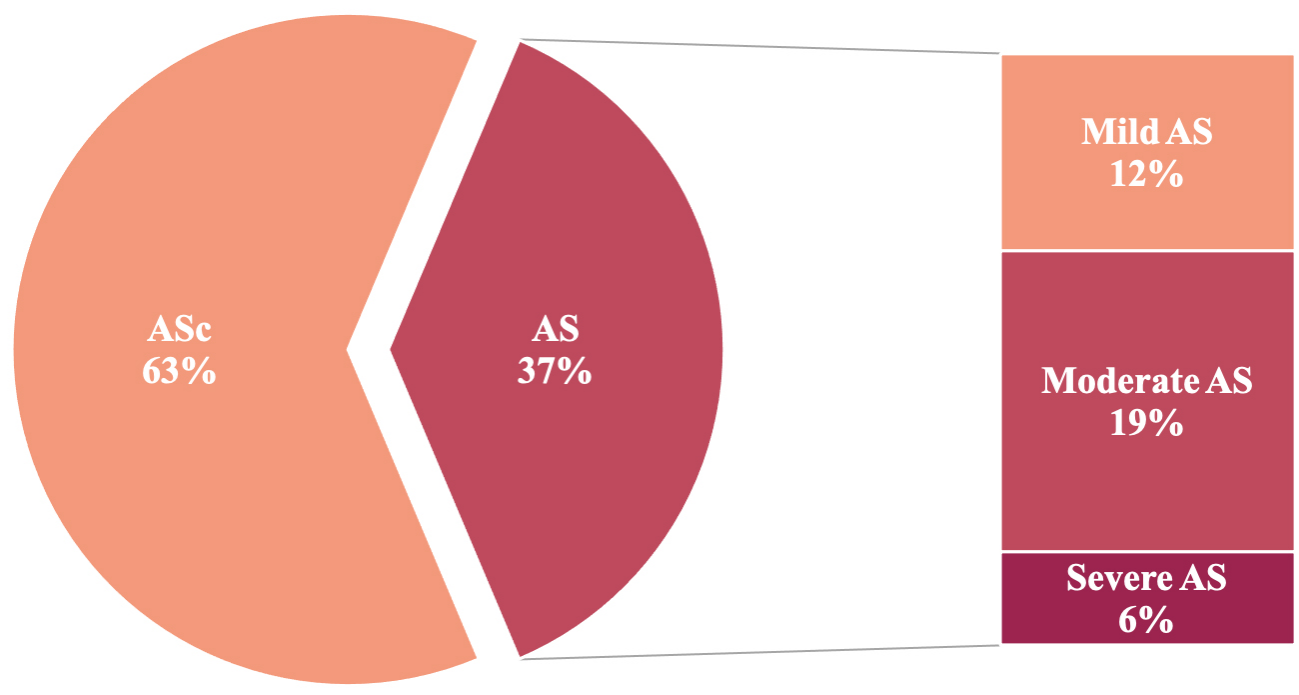
**Prevalence of CAVD progression in patients with preexistent aortic valve sclerosis. **According to the follow-up echocardiography 96 (63%) patients showed stable ASc, whereas 57 (37%) of the study patients experienced progression of CAVD; 12.4% of the patients developed mild AS, 19.0% showed moderate AS, and 5.9% suffered from severe AS. AS, aortic stenosis; ASc, aortic valve sclerosis; CAVD, calcific aortic valve disease.

The mean time to follow-up was 1463 ± 953 days. At follow-up echocardiography, the mean AV Vmax of the overall study population was 2.3 ± 0.8 m/s, the MPG was 12.1 ± 9.8 mmHg and the mean AVA was 1.6 ± 0.8 ${\mathrm{cm}}^{2}$. Out of 153 patients, approximately one-third developed AS with a mean AV Vmax of 3.2 ± 0.5 m/s, whereas 96 patients (63%) showed stable ASc with a mean AV Vmax of 1.7 ± 0.3 m/s. In detail, 12.4% of the patients developed mild AS, 19.0% showed moderate AS and 5.9% suffered from severe AS, as demonstrated in Fig. 2.

### 3.2 Clinical Parameters According to Calcific Aortic Valve Disease Progression

Clinical parameters of the two CAVD groups (AS vs. ASc) are presented in Table 2. The AS group was younger (70.1 ± 10.5 years vs. 75.0 ± 6.0 years; *p* = 0.001) and presented with higher rates of CKD (35.1% vs. 17.7%; *p* = 0.01) and dialysis-dependent kidney insufficiency (10.5% vs. 2.1%; *p* = 0.02) at baseline. Other known risk factors for the development of cardiovascular diseases such as arterial hypertension (*p* = 0.93), diabetes (*p* = 0.34), dyslipidemia (*p* = 0.06) or smoking status (*p* = 0.11) were not significantly associated with CAVD progression. At both time points, the ASc group showed a more dilated ascending aorta than the AS group (baseline: 2.6 ± 0.3 vs. 2.8 ± 0.4; *p* < 0.001; follow-up: 2.6 ± 0.4 vs. 2.9 ± 0.4; *p* = 0.001). Both the treatment with oral anticoagulant drugs (*p* = 0.25) and anti-platelet agents (mono antiplatelet therapy (MAPT): *p* = 0.52; dual antiplatelet therapy (DAPT): *p* = 0.09) was not associated with CAVD progression.

**Table 2.** **Clinical and echocardiographic parameters according to CAVD progression**.

		AS group	ASc group	*p*-value
		(n = 57)	(n = 96)	
Clinical parameters				
Age, ± SD		70.1 ± 10.5	75.0 ± 6.0	**0.001**
BMI, ± SD		26.9 ± 5.4	27.0 ± 4.1	0.41
Male sex, n (%)		41 (71.9)	61 (63.5)	0.29
PAD, n (%)		8 (14.0)	11 (11.5)	0.64
CKD, n (%)		20 (35.1)	17 (17.7)	**0.015**
Dialysis, n (%)		6 (10.5)	2 (2.1)	**0.023**
Hypertension, n (%)		49 (86.0)	83 (86.5)	0.93
Diabetes, n (%)		15 (26.3)	19 (19.8)	0.34
Dyslipidemia, n (%)		27 (47.4)	60 (39.2)	0.06
Smoker, n (%)		22 (38.6)	25 (26.3)	0.11
Atrial fibrillation, n (%)		26 (45.6)	50 (52.1)	0.44
History of CAD, n (%)		31 (54.4)	59 (61.5)	0.39
Previous stroke, n (%)		8 (14.0)	7 (4.6)	0.17
MAPT, n (%)		17 (30.4)	34 (35.4)	0.52
DAPT, n (%)		13 (22.8)	12 (12.5)	0.09
OAC/DOAC, n (%)		26 (45.6)	53 (55.2)	0.25
Echocardiographic parameters at baseline				
LVOT diameter, cm		2.2 ± 0.3	2.1 ± 0.2	**0.037**
Aortic root diameter, cm		3.0 ± 0.4	3.0 ± 0.3	0.32
Ascending aorta diameter, cm		2.6 ± 0.3	2.8 ± 0.4	< **0.001**
Cusp thickness NCC, cm		0.31 ± 0.06	0.24 ± 0.05	< **0.001**
Cusp thickness LCC, cm		0.29 ± 0.06	0.23 ± 0.05	< **0.001**
Cusp thickness RCC, cm		0.33 ± 0.07	0.24 ± 0.05	< **0.001**
AVA plan., ${\mathrm{cm}}^{2}$		1.7 ± 0.5	2.2 ± 0.5	< **0.001**
AVA by continuity equation., ${\mathrm{cm}}^{2}$		1.7 ± 0.7	2.2 ± 0.6	0.017
MPG, mmHg		8.7 ± 3.3	5.5 ± 2.0	< **0.001**
maxPG, mmHg		16.6 ± 5.6	10.9 ± 4.4	< **0.001**
Vmax, m/s		1.9 ± 0.3	1.6 ± 0.32	< **0.001**
Time to peak velocity, ms		94.2 ± 26.0	88.8 ± 25.1	0.10
Stroke volume, mL		55.6 ± 18.4	57.2 ± 40.7	0.39
Systolic duration, sec		0.3 ± 0.04	0.29 ± 0.04	0.12
Aortic regurgitation, n (%)				< **0.001**
	- Grade 0	25 (43.9)	46 (47.9)	
	- Grade I	21 (36.8)	44 (45.8)	
	- Grade II	11 (19.3)	6 (6.3)	
	- Grade III	-	-	
Calcification NCC, n (%)		25 (43.9)	20 (20.8)	**0.003**
Calcification LCC, n (%)		28 (49.1)	11 (11.5)	< **0.001**
Calcification RCC, n (%)		44 (78.6)	27 (28.4)	< **0.001**
Calcification anulus, n (%)		48 (84.2)	94 (97.9)	**0.002**
Calcification leaflet tips, n (%)		48 (84.2)	85 (88.5)	0.44
Reduced mobility NCC, n (%)		14 (24.6)	4 (4.2)	< **0.001**
Reduced mobility LCC, n (%)		10 (17.5)	2 (2.1)	< **0.001**
Reduced mobility RCC, n (%)		16 (28.6)	8 (8.3)	< **0.001**
Mitral regurgitation, n (%)				0.60
	- Grade 0	7 (12.3)	8 (8.3)	
	- Grade I	30 (52.6)	61 (63.5)	
	- Grade II	18 (31.6)	24 (25.0)	
	- Grade III	2 (3.5)	3 (3.1)	
Heart rate, bpm		73 (64/87.2)	68 (60/81.7)	0.17
LV hypertrophy, n (%)		31 (54.4)	54 (56.3)	0.682
IVSd, cm		1.3 ± 0.3	1.3 ± 0.3	0.37
IVSs, cm		1.6 ± 0.3	1.6 ± 0.3	0.45
E/e’, ± SD		16.3 (10.8/23.3)	12.2 (10.2/19.0)	0.08
Ejection fraction, %		52.3 (12.2)	54.5 (11.5)	0.13
LVEDV, mL		101.0 (86.8/126.9)	101.4 (75.2/117.8)	0.13
LVESV, mL		47.9 (39.4/58.6)	47.8 (28.9/58.6)	0.044
LA volume end-diastolic, mL		37.9 (22.1/70.1)	35.6 (22.2/63.8)	0.69
LA volume end-systolic, mL		60.6 (41.2/89.5)	55.5 (41.2/84.9)	0.54
Diastolic dysfunction, n (%)				0.46
	- Grade 0	20 (35.7)	33 (34.4)	
	- Grade I	24 (42.9)	33 (34.4)	
	- Grade II	5 (8.9)	17 (17.7)	
	- Grade III	7 (12.5)	13 (13.5)	
Echocardiographic parameters at follow-up				
Time to follow-up, days		1547 ± 996	1414 ± 929	0.40
Aortic stenosis, n (%)				< **0.001**
	- None	-	96 (100)	
	- Mild	19 (33.3)	-	
	- Moderate	29 (50.9)	-	
	- Severe	9 (5.9)	-	
LVOT diameter, cm		2.1 ± 0.3	2.1 ± 0.3	0.06
Aortic root diameter, cm		3.0 ± 0.4	3.0 ± 0.4	0.47
Aortic ascendens diameter, cm		2.6 ± 0.4	2.9 ± 0.4	**0.001**
Cusp thickness NCC, cm		0.38 ± 0.09	0.27 ± 0.06	< **0.001**
Cusp thickness LCC, cm		0.37 ± 0.08	0.25 ± 0.06	< **0.001**
Cusp thickness RCC, cm		0.40 ± 0.08	0.27 ± 0.06	< **0.001**
AVA plan., ${\mathrm{cm}}^{2}$		0.9 ± 0.4	2.0 ± 0.5	< **0.001**
AVA by continuity equation., ${\mathrm{cm}}^{2}$		1.0 ± 0.3	2.1 ± 0.6	< **0.001**
MPG, mmHg		22.4 ± 8.9	5.9 ± 2.4	< **0.001**
maxPG, mmHg		42.9 ± 14.3	13.2 ± 12.4	< **0.001**
Vmax, m/s		3.2 ± 0.5	1.7 ± 0.3	< **0.001**
Time to peak velocity, ms		98.2 ± 25.9	84.4 ± 24.3	< **0.001**
Stroke volume, mL		53.3 ± 20.0	54.7 ± 20.6	0.69
Systolic duration, sec		0.3 ± 0.04	0.3 ± 0.05	0.16
Aortic regurgitation, n (%)				0.14
	- Grade 0	42 (43.8)	25 (43.9)	
	- Grade I	49 (51.0)	24 (42.1)	
	- Grade II	8 (14.0)	5 (5.2)	
	- Grade III	-	-	
Calcification NCC, n (%)		44 (77.2)	35 (36.5)	< **0.001**
Calcification LCC, n (%)		49 (86.0)	26 (27.1)	< **0.001**
Calcification RCC, n (%)		51 (91.1)	46 (48.4)	< **0.001**
Calcification anulus, n (%)		56 (98.2)	93 (96.9)	0.61
Calcification leaflet tips, n (%)		55 (96.5)	88 (91.7)	0.24
Reduced mobility NCC, n (%)		42 (73.7)	8 (8.3)	< **0.001**
Reduced mobility LCC, n (%)		35 (61.4)	4 (4.2)	< **0.001**
Reduced mobility RCC, n (%)		47 (83.9)	20 (20.8)	< **0.001**
Mitral regurgitation, n (%)				**0.012**
	- Grade 0	6 (10.5)	2 (2.1)	
	- Grade I	30 (52.6)	63 (65.6)	
	- Grade II	18 (31.6)	31 (32.3)	
	- Grade III	3 (5.3)	-	
Heart rate, bpm		70 (63.5/76)	68 (58.2/81)	0.19
LV hypertrophy, n (%)		44 (77.2)	57 (59.4)	**0.024**
IVSd, cm		1.4 (0.3)	1.3 (0.3)	**0.009**
IVSs, cm		1.7 (0.3)	1.6 (0.3)	0.08
Ee’, ± SD		16.5 (11.9/23.9)	15.3 (11/23.6)	0.20
Ejection fraction, %		52.6 (13.5)	55.6 (10.4)	0.07
LVEDV, mL		96.6 (76.6/130.2)	96.7 (72.4/122.9)	0.59
LVESV, mL		44.9 (28.0/65.2)	40.8 (29.5/56.1)	0.53
LA volume (end-diastolic), mL		55.4 (32.0/70.9)	40.3 (25.4/64)	0.06
LA volume (end-systolic), mL		70.7 (45.5/97.5)	62.2 (46.3/82.6)	0.14
Diastolic dysfunction, n (%)				0.12
	- Grade 0	19 (33.3)	24 (25.0)	
	- Grade I	25 (43.9)	32 (33.3)	
	- Grade II	4 (7.0)	16 (16.7)	
	- Grade III	9 (15.8)	24 (25.0)	

Values are displayed as mean (± SD), median (IQR 1/3) or n (%). Statistical significance is highlighted in bold. AS, aortic stenosis; ASc, aortic valve sclerosis; AVA, aortic valve area; AVA plan., aortic valve area plane; BMI, body mass index; CAD, coronary artery disease; CKD, chronic kidney disease; DAPT, dual antiplatelet therapy; DOAC, direct oral anticoagulant; E/e’, early mitral inflow velocity to mitral annular early diastolic velocity ratio; IVSd, diastolic interventricular septal thickness; IVSs, systolic interventricular septal thickness; LA, left atrial; LCC, left-coronary cusp; LV, left ventricular; LVEDV, left ventricular end-diastolic volume; LVESV, left ventricular end-systolic volume; LVOT, left ventricular outflow tract; MAPT, mono antiplatelet therapy; maxPG, maximum pressure gradient; MPG, mean pressure gradient; NCC, non-coronary cusp; OAC, oral anticoagulant; PAD, peripheral artery disease; RCC, right-coronary cusp; Vmax, peak flow velocity; CAVD, calcific aortic valve disease; IQR, interquartile range.

### 3.3 Echocardiographic Parameters According to Calcific Aortic Valve Disease Progression

Baseline and follow-up echocardiographic parameters according to the CAVD groups are shown in Table 2. Patients with CAVD progression (i.e., AS group) presented with a mildly, but significantly elevated AV Vmax (AS group: 1.9 ± 0.3 m/s versus ASc group: 1.6 ± 0.3 m/s; *p* < 0.001), maxPG (AS group: 16.6 ± 5.6 mmHg versus ASc group: 10.9 ± 4.4 mmHg; *p* <0.001), and MPG (AS group: 8.7 ± 3.3 mmHg versus ASc group: 5.5 ± 2.0 mmHg; *p* < 0.001), at baseline. Patients in this group displayed significantly higher rates of major calcification (*p* < 0.001), advanced thickening (*p* < 0.001) of the valve cusps (**Supplementary Fig.1**), and showed a reduced mobility of the LCC, RCC and NCC. Furthermore, the AS group had significantly higher rates of concomitant advanced aortic valve regurgitation (AR) at baseline (AR grade II: 19.3% vs. 6.3; *p* < 0.001).

**Fig. 3.**
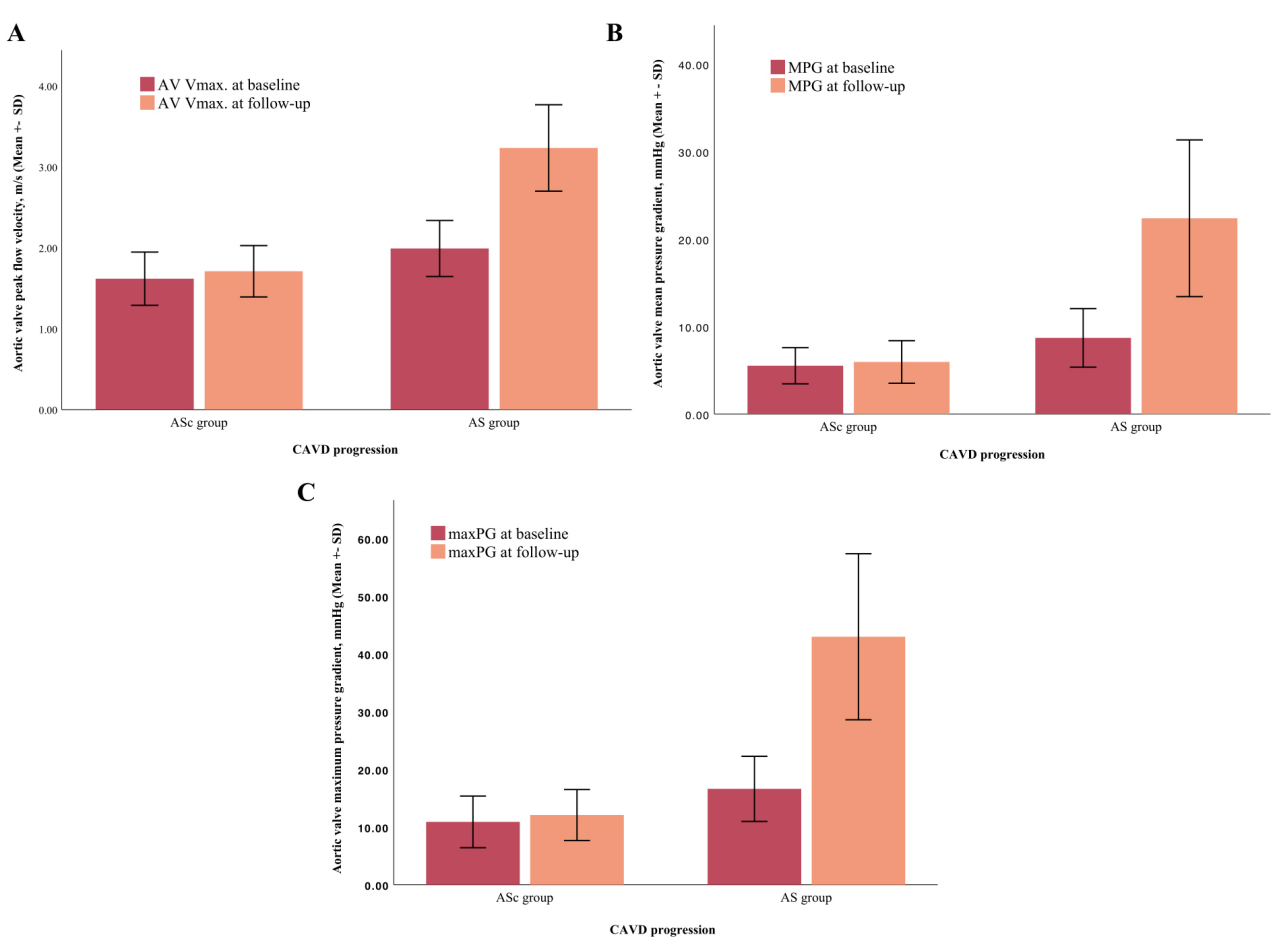
**Comparison between echocardiographic parameters at baseline and follow-up in accordance with CAVD progression. **The AV Vmax (A), MPG (B) and maxPG (C) increased significantly within the follow-up period of 4 years in the AS group. CAVD, calcific aortic valve disease; AS, aortic stenosis; ASc, aortic valve sclerosis; AV Vmax, aortic valve peak flow velocity; maxPG, maximum pressure gradient; MPG, mean pressure gradient; SD, standard deviation.

At follow-up echocardiography, 19 patients (33.3%) had mild AS, 29 patients (50.9%) presented with moderate AS and 9 patients (5.9%) suffered from severe AS (Fig. 2). The mean time to follow-up did not differ between the CAVD groups (AS group: 1547 ± 996 days vs. ASc group: 1414 ± 929 days; *p* = 0.4). In the AS group, the average MPG was 22.4 ± 8.9 mmHg, the maxPG was 42.9 ± 14.3 mmHg and the mean AV Vmax was 3.2 ± 0.5 m/s in the follow-up echocardiography. In the ASc group, the average MPG was 5.9 ± 2.4 mmHg, the maxPG was 13.2 ± 12.4 mmHg and the mean AV Vmax was 1.7 ± 0.3 m/s. A direct comparison between these parameters at baseline and follow-up is shown in Fig. 3.

### 3.4 Multivariate Regression Analysis

To identify independent predictors for disease progression, we performed a multivariate regression analysis, which included univariate predictors with a *p*-value < 0.05, as shown in Table 3. In the univariate regression analysis, CKD (*p* = 0.017), dialysis-dependent kidney insufficiency (*p* = 0.04), moderate aortic valve regurgitation (*p* = 0.03), major aortic valve calcification (*p* < 0.001), reduced valve motion (*p* < 0.001) and valve cusp thickness >0.26 cm (*p* < 0.001) were associated with CAVD progression. The multivariate analysis identified major valve calcification (hazard ratio [HR]: 4.8, 95% confidence interval [CI]: 1.5–15.9; *p* = 0.009) and valve thickness >0.26 cm (HR: 16.6, 95% CI: 5.4–50.7; *p* < 0.001) at baseline as independent predictors for the development of AS. CKD (*p* = 0.06), dialysis-dependent kidney insufficiency (*p* = 0.19), moderate aortic valve regurgitation (*p* = 0.5) and reduced valve motion (*p* = 0.15) were not independently associated with disease progression.

**Table 3.** **Multivariate analysis**.

	Univariate analysis	*p* value	Multivariate analysis	*p* value
	HR (95% CI)		HR (95% CI)	
Male sex	2.8 (0.7–2.9)	0.28	-	-
Chronic kidney disease	2.5 (1.8–5.3)	**0.017**	3.3 (0.9–11.8)	0.06
Dialysis	5.5 (1.0–28.3)	**0.04**	5.5 (0.4–70.6)	0.19
Ejection fraction	0.9 (0.9–1.1)	0.25	-	
Diabetes	1.4 (0.6–3.1)	0.35	-	-
PAD	1.2 (0.4–3.3)	0.64	-	-
Atrial fibrillation	0.8 (0.4–1.4)	0.43	-	-
Arterial hypertension	0.9 (0.4–2.5)	0.93	-	-
Nicotine abuse	1.7 (0.8–3.5)	0.11	-	-
Dyslipidemia	0.5 (0.3–1.0)	0.07	-	-
Moderate aortic regurgitation	3.3 (1.1–10.2)	**0.03**	1.6 (0.3–2.6)	0.5
Cusp thickness >0.26 cm	23.2 (9.2–58.5)	< **0.001**	16.6 (5.4–50.7)	< **0.001**
Major valve calcification	13.9 (5.1–38.0)	< **0.001**	4.8 (1.5–15.9)	**0.009**
Reduced valve motion	6.1 (2.8–13.5)	< **0.001**	2.0 (0.7–5.8)	0.15

Statistical significance is highlighted in bold. CI, confidence interval; HR, hazard ratio; PAD, peripheral artery disease.

### 3.5 Receiver Operating Characteristics Curve Analysis

**Fig. 4.**
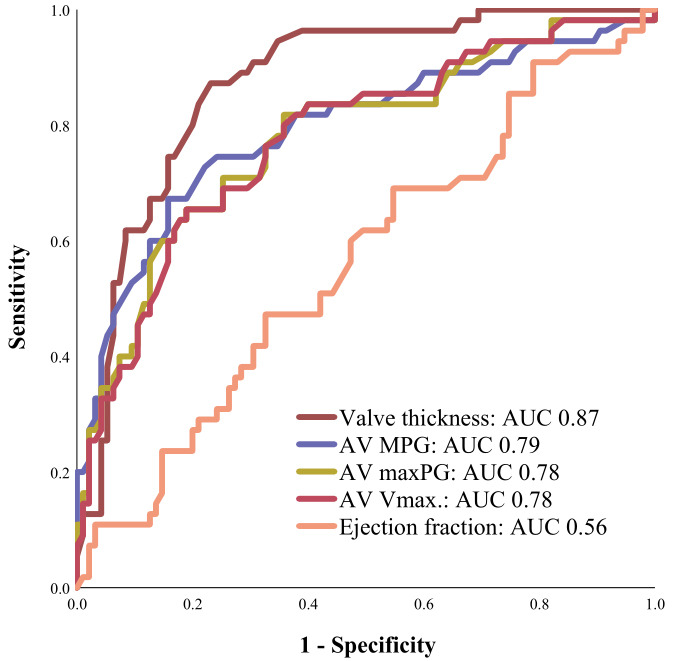
**Receiver Operating Characteristics (ROC) curve analysis.** Advanced valve thickness showed the strongest association with disease progression in ROC curve analysis. AUC, area under the curve; AV Vmax, aortic valve peak flow velocity; maxPG, maximum pressure gradient; AV, aortic valve; MPG, mean pressure gradient.

In a receiver operating characteristics curve analysis, comparing the predictive value of the different echocardiographic parameters for disease progression, advanced valve thickness (area under the curve [AUC]: 0.87, 95% CI: 0.81–0.93, *p* < 0.001) showed the strongest association with disease progression, as presented in Fig. 4.

## 4. Discussion

In this study including 153 patients with visual signs of ASc but without AS, we assessed echocardiographic parameters to evaluate the prevalence and the progression of CAVD to identify of patients at high risk of developing aortic valve stenosis. The main results of our study are as follows:

Out of 153 study patients, 1/3 experienced progression of CAVD,

Calcification and advanced thickness of the valve cusps >0.26 cm were significantly associated with disease progression and independent predictors for the development of AS.

### 4.1 Prevalence of Aortic Valve Sclerosis and Disease Progression

ASc, the preceding stage of CAVD, displays focal areas of valve calcification and leaflet thickening without functional relevant obstruction of the left ventricular outflow tract [8]. It is one of the most frequent findings in transthoracic echocardiography with a growing incidence in the older population [9]. ASc has been reported to be present in almost 30% of adults aged over 65 years [4, 10], whereas the prevalence of disease progression from ASc to AS differs in the literature. One of the largest prospective studies included >2000 patients with ASc and a mean age of 69 years, of whom 16% developed AS within 8 years of follow-up; 10.5% developed mild stenosis, 3% advanced to moderate stenosis and 2.5% progressed to severe aortic stenosis [11]. Interestingly, a meta-analysis of twenty-two studies revealed a progression rate of 1.8-1.9% of patients per year in individuals with baseline ASc [8]. Faggiano *et al*. [9] found a progression rate from ASc to any degree of AS in 32.7% of patients in a smaller cohort of 400 individuals with a mean age of 68 years, during a follow-up period of 4 years; 2.5% of the patients developed severe AS, 5.2% proceeded to moderate AS and 25% displayed mild AS. Comparable results could be observed in our study. We found a progression rate of 37% within 4 years of follow-up in a cohort of 153 patients with a mean age of 73 years; 12% of patients developed mild AS, 19% presented with moderate AS and 6% progressed to severe AS. Despite the high prevalence of CAVD and its clinical implications, we are currently still not able to interrupt the vicious circle of AV inflammation and calcification in order to delay or prevent disease progression, due to the absence of any pharmacological treatment option.

### 4.2 Calcific Aortic Valve Disease and Comorbidities

Several studies have already evaluated the overlap of traditional cardiovascular risk factors (CRF) and the presence of aortic valve calcification [12, 13, 14, 15, 16]. In the past, comorbidities such as advanced age, male gender, arterial hypertension, dyslipidemia and smoking have been shown to be associated with the development of aortic valve calcification and atherosclerotic disease to a comparable degree [12, 17], supporting the hypothesis that both diseases have a shared pathomechanistic processes. These data are supported by our study results, as we observed a high prevalence of CRF and concomitant coronary artery disease at baseline in our study population. On the basis of a prospective analysis, including 70 patients with baseline aortic valve calcification, Messika-Zeitoun *et al*. [18] were able to show that the progression of established ASc was unrelated to cardiovascular risk factors, age and sex. Bellamy *et al*. [19] evaluated the association between CAVD progression and cholesterol levels at baseline in a cohort of 156 patients revealing no significant correlation between blood cholesterol concentrations and the progression of ASc. Corroborating results have been described by other major prospective studies including SEAS (Simvastatin and Ezetimibe in Aortic Stenosis), SALTIRE (Scottish Aortic Stenosis and Lipid Lowering Trial, Impact on Regression), ASTRONOMER (Effect of Cholesterol Lowering on the Progression of Aortic Stenosis in Patients With Mild to Moderate Aortic Stenosis). These trials could not find a relationship between LDL levels and progressive aortic valve disease on the one hand, and were not able to confirm the beneficial effect of statins on CAVD progression, on the other [20, 21, 22].

In our study, CKD and terminal dialysis-dependent renal insufficiency were the only clinical factors, that were significantly associated with disease progression. This result is not surprising, since CKD and especially long-term dialysis are often linked with the occurrence of cardiovascular events. Interestingly, patients with CAVD progression were significantly younger with a mean age of 70 years at baseline compared to patients with stable ASc, who were five years older on average. Higher rates of other traditional CRF were not significantly associated with disease progression. This result should nevertheless be considered cautiously, since our study is based on a retrospective analysis of a small sample population.

Our study results showed that the ASc group presented a more dilated ascending aorta compared to the AS group at baseline and follow-up. This result might be explained by the higher rates of CKD and dialysis in the ASc group, but also by the significantly older age of the ASc group, representing a known risk factor for the development of aortic dilatation and aneurysms.

Larger prospective studies are needed to identify clinical risk factors associated with disease progression, to pave the way for the development of targeted therapies.

### 4.3 Transthoracic Echocardiography and Calcific Aortic Valve Disease

The reliable and early identification of patients with ASc, who are at high risk of developing AS, should be another important goal in AV research. In this context, imaging techniques play an important role. Transthoracic echocardiography (TTE) is the gold standard for the evaluation of CAVD and the quantification of AS severity. Beside the visual assessment of the leaflet anatomy and the extent of valve calcification, the evaluation of functional parameters are pivotal during diagnostic work-up [23, 24].

In our study, we evaluated echocardiographic parameters with regard to their forecast value to predict the development of AS and identified degree of calcification, valve thickening and reduced valve motion to be associated with CAVD progression. In the multivariate analysis, major calcification and valve thickness >0.26 cm were independent predictors for the development of AS. However, one must be aware that the reliable and accurate identification of aortic valve calcification using echocardiography is still challenging, given the variability of scanner settings, image quality and the examiner’s experience. In our study, the quantification of ASc based on visual assessment, as a precise and objective quantification of aortic valve sclerosis in the early stage, is nearly impossible due to the limited resolution of current ultrasound scanners. The only alternative to quantify aortic valve sclerosis more precisely would be via examination by computed tomography [25], which unfortunately would be accompanied by high radiation exposure, especially if repetitive examinations are needed. The huge advantage of the assessment of visual echocardiographic parameters, as described above, is the simple, non-invasive, cost-effective and radiation-free image acquisition, which could be performed easily in every routine TTE examination. As a consequence, patients with ASc and visual signs of advanced calcification and valve thickening, could be closely monitored with regard to echocardiographic signs of disease progression in conjunction with the onset of new symptoms.

### 4.4 Limitations

The assessment and quantification of aortic valve calcification with transthoracic echocardiography represents a major limitation of our study, as precise and objective measurements with this examination method are nearly impossible. In our study, the quantification of aortic valve calcification as a binary parameter is based on visual estimations and thus represents a subjective and examiner-dependent parameter. Cardiac computed tomography scans would have been needed to objectively quantify the degree of valve calcification and to confirm our results. Therefore, the results of this study should be considered hypothesis-generating. Prospective and larger trials are necessary to confirm our results.

## 5. Conclusions

One-third of patients with aortic valve sclerosis at baseline progressed to (any degree of) AS within a follow-up period of four years. Advanced aortic valve calcification and a cusp thickness >0.26 cm at baseline echocardiography were independent predictors for the development of AS in these patients. The acquisition of simple echocardiographic parameters can help to identify patients at a particularly high risk to develop aortic valve stenosis.

## Data Availability

The dataset generated and analyzed during the current study is available from the corresponding author on reasonable request.

## Supplementary Material

Supplementary material associated with this article can be found, in the online version, at https://doi.org/10.31083/j.rcm2410293.
