# Supplementary material for: Echocardiographic Progression of Calcific Aortic Valve Disease in Patients with Preexisting Aortic Valve Sclerosis
Source: Rev Cardiovasc Med. 2023 Oct 17;24(10):293. doi: 10.31083/j.rcm2410293 (PMC11273110; doi:10.31083/j.rcm2410293)
Supplement: Supplementary file 1 [file 2153-8174-24-10-293-s1.docx]

**Supplementary Material**


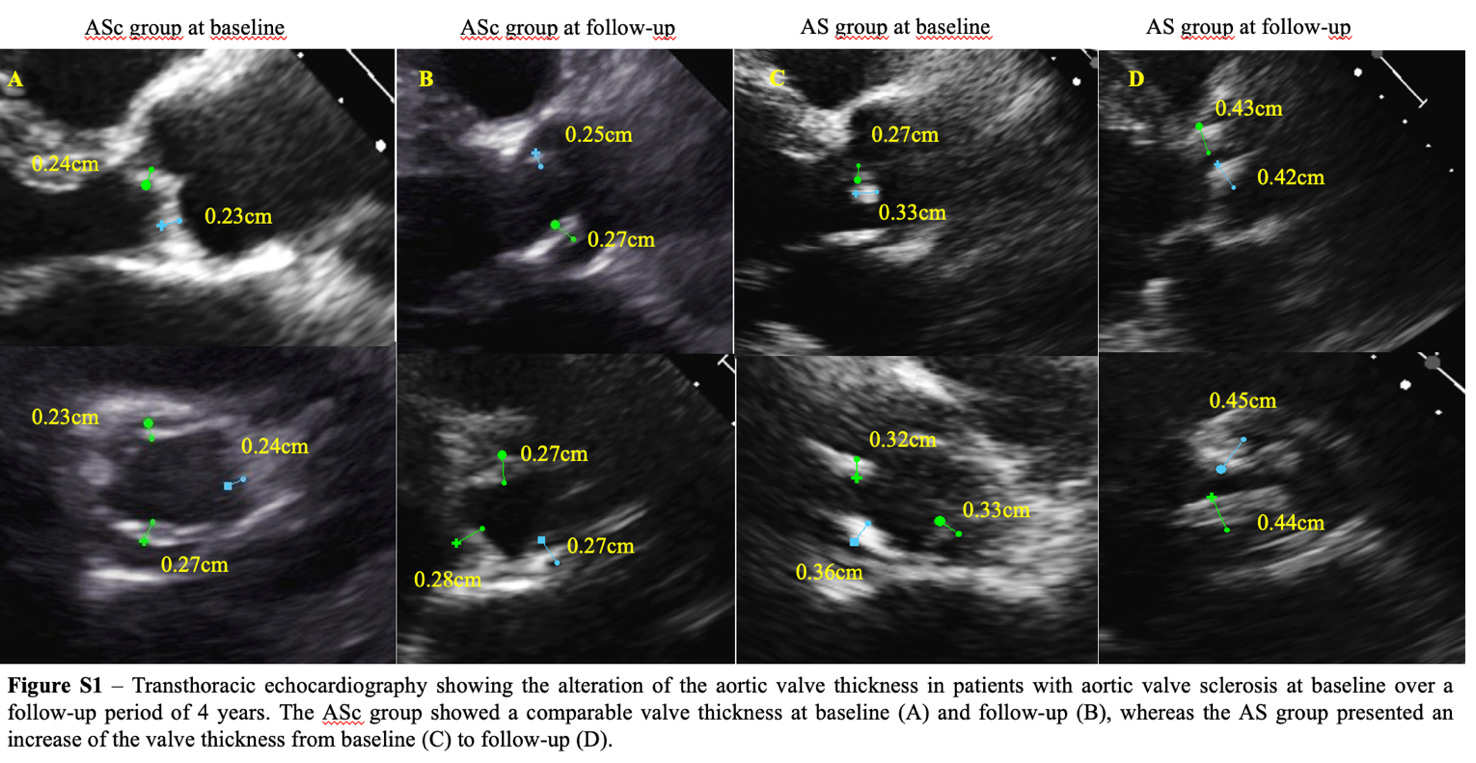


**Supplementary Fig. 1. Transthoracic echocardiography showing the alteration of the aortic valve thickness in patients with aortic valve sclerosis at baseline and over a follow-up period of 4 years.**
